# The Crucial Role of Rotation Speed on the Determination of Tafel Slopes of Electrocatalysts in Rotating Disk Electrode Experiments

**DOI:** 10.1021/acselectrochem.5c00210

**Published:** 2025-07-24

**Authors:** Felix Hiege, Luca Marie Sicking, Kannasoot Kanokkanchana, Paolo Cignoni, Victor Dudarev, Alfred Ludwig, Kristina Tschulik

**Affiliations:** † Faculty of Chemistry and Biochemistry, Chair of Analytical Chemistry II, 9142 Ruhr University Bochum, Universitätsstraße 150, 44801 Bochum, Germany; ‡ 28272 Max-Planck-Institut für Nachhaltige Materialien GmbH, Max-Planck-Straße 1, 40237 Düsseldorf, Germany; § Materials Discovery and Interfaces, Institute for Materials, 153605 Ruhr University Bochum, Universitätsstraße 150, 44801 Bochum, Germany

**Keywords:** Reaction Kinetics, Oxygen Evolution Reaction, Rotating Disk Electrode, Nickel Selenide, Tafel Analysis

## Abstract

This article highlights the crucial influence of rotation speeds in determining the Tafel slopes of electrocatalysts in rotating disk electrode (RDE) experiments. As an exemplary system, we investigate catalytically active nickel oxide/oxyhydroxide films that were generated from the transformation of electrodeposited nickel selenide at Au-RDEs, under oxygen evolution reaction (OER) conditions in alkaline electrolytes at three different rotation rates (1600, 2500, and 4000 rpm). Tafel slopes were extracted using cyclic voltammetry (CV) and chronoamperometry (CA). Our results underline the benefit of higher rotation speed in overcoming undesired mass transport limitations. Additionally, we point out that using CA instead of CV methods is preferable for the extraction of kinetic parameters since CA ensures steady state conditions and simplifies data analysis. Furthermore, we highlight that rigorous determination of reaction mechanisms based on Tafel slopes is only appropriate if the boundary conditions of the Butler-Volmer formalism are fulfilled, and we recommend not using Tafel slopes as the single parameter to compare the activity of electrocatalysts if these are obtained at different experimental conditions. Instead, we suggest reporting relevant measurement parameters such as the rotation speed of the experiment, way of Ohmic drop compensation, electrode geometry, and the electrochemical method used for Tafel analysis alongside the calculated Tafel slopes. Doing so may allow comparison to literature-reported Tafel slopes if the data were determined under similar experimental conditions. Rather than focusing on individual values, trends between systematically modified catalysts or at different aging stages should be considered meaningful.

## Graphical abstract

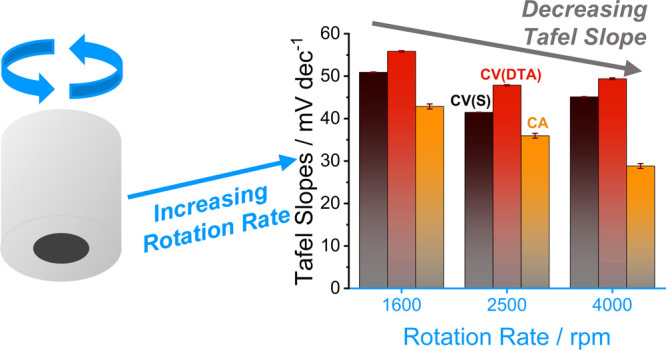

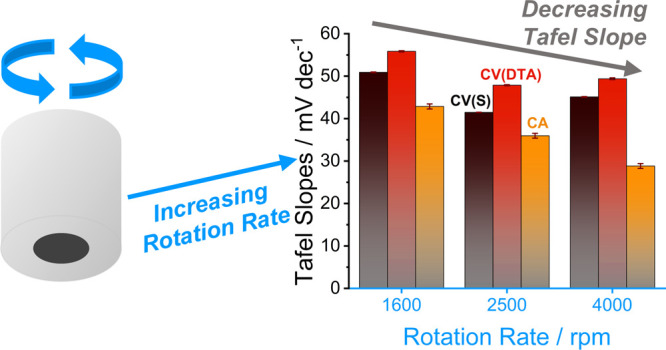

## Introduction

In recent years, plenty of work has been invested in determining and interpreting Tafel slopes, especially to investigate reaction mechanisms and evaluate the kinetic activity of electrocatalysts for kinetically sluggish reactions, e.g., the oxygen evolution reaction (OER).^1–5^ Even though the experimental conditions in terms of cell and electrode geometries, electrochemical surface areas (ECSA) of the catalysts,^6,7^ or electrochemical procedures deviate greatly in some cases, Tafel slopes are widely used in the literature as a key parameter to describe the activity of a catalyst system.^8–12^ The kinetic current density *j*_k_ in the Tafel equation is given by the sum of the anodic *j*_a_ and cathodic *j*_c_ current density contributions^3,13^ which can be simplified at high overpotentials, to be fully dominated by one of the two partial current densities.^1,3,14,15^ For example, when investigating the OER, *j*_a_ is dominating the overall current at high anodic overpotentials. The overpotential *η* reflects the potential difference between the applied potentials *E* and the equilibrium potential *E*_eq_ of a reaction:

$$\eta =E-E_{\mathrm{eq}}$$ 1

For the OER, *E*_eq,OER_ = 1.23 V vs. RHE.^15–17^ The kinetic current density *j*_k_ at high overpotentials is expressed by

$$j_{k}=j_{0}e^{-\alpha f\eta }$$ 2

There *f* = *RT/F*, *R* is the universal gas constant, *T* is the temperature, *F* is the Faraday constant, and *j*_0_ is the exchange current density.^1^ In Equation 3, the logarithmic form of Equation 2, the charge transfer coefficient *α* is accessible from slope *b* in the linear region of the plot, and the exchange current density *j*_0_ from the intercept of the linear fit with the abscissa.^15,17^ Notably, ideal Tafel behavior as described by Equation 3 refers to the high overpotential limit, that is, a fully irreversible reaction under the applied experimental conditions. As a rule of thumb, this is reached when the current contribution of the back reaction is less than ≈1%,^15^ which is fulfilled for the oxygen reduction reaction in all potential windows considered for our Tafel analyses (>1.52 V vs. RHE with *η* at 10 mA cm^–2^ of >290 mV in all experiments, see Figure 2 and Figure S5).

$$\log\;\left|j_{k}\right|=-\frac{\alpha F}{2.303RT}\eta +j_{0}=b\eta +j_{0}$$ 3

From Equation 3 it can also be understood that facile electrochemical reaction kinetics are described by high *j*_0_ *and* low Tafel slopes.^1,14,15,17^

However, the utilization of Tafel slopes to describe the charge transfer kinetics, or identifying rate-determining steps of an electrochemical reaction is only appropriate if the Butler-Volmer (BV) model can be applied to this system. This implies that the system is probed at steady state conditions, where the charge coefficient *α* ^5,15,17^ and surface concentrations of reactants and intermediates are constant.^13,18^ Furthermore, the current must not be affected by non-Faradaic contributions, such as capacitive currents from double-layer charging,^3,19,20^ or mass transport limitations.^1,2,19,21^ The latter is usually fulfilled if experimental currents are less than 10% of the limiting current density.^15,17^ For larger currents, the kinetic current density *j*_k_ can be calculated from the experimentally determined current density *j* by correcting mass transport limitations using the Levich equation.^1,3^ Noteworthy, Tafel analysis requires accurate correction of the Ohmic drop as well.^1,14,18,22^ Lastly, it should be noted that for reactions involving multiple electron transfer steps, like OER, in which four electrons are exchanged, the slowest one will determine the overall reaction rate.

To demonstrate the crucial effect of the experimental conditions (including mass transport limitations) on the determination of Tafel slopes, we investigate electrodeposited nickel-selenide-based films on Au-RDEs and extract Tafel slopes from electrochemical experiments performed at three different rotation rates (1600, 2500, 4000 rpm). Nickel selenides are state-of-the-art precious metal-free pre-catalyst materials for OER in alkaline electrolytes.^23–25^ Thus, an electrochemical pre-treatment (SI-Section 1.4) is conducted to facilitate the transformation into the catalytically active oxyhydroxide species.^26–29^ Since several reports highlight the effect of trace amounts of iron on the catalytic activity of the nickel oxyhydroxide catalysts,^2,30–34^ we ensure that iron concentration in the electrolyte is controlled and constant throughout the study. Cyclic voltammetric (CV) and chronoamperometric (CA) methods were used to monitor experimental currents, which were normalized by the geometric area of the electrode and mass transport corrected using the limiting current estimated from the Koutecký-Levich correction (see SI-Section 1.5 for details). Besides standard Tafel analysis, differential Tafel plots have emerged in recent years as a useful approach to improve the quality of the current data by reducing the influence of non-exponential current contributions and identifying accurate potential ranges for Tafel analysis.^1,3,8^ Therefore, we extract Tafel slopes from CV experiments in this study via both standard (STA) and differential (DTA) Tafel plots (using the 1^st^ derivative of the kinetic current).

## Experimental Section

Detailed descriptions of the experimental conditions are provided in the SI. Electrodepositions of nickel selenides were conducted according to the procedure of Cao et al.^25^ at -0.79 V vs. Ag|AgCl|3 M KCl under coulometric termination control (76 mC cm^–2^_geo_) on gold supports. All OER experiments were conducted in 0.1 M KOH + 1.0 mM Fe­(NO_3_)_3_. For the RDE experiments, either an MRS (AFMSRCE, Pine Research) or an ALS (RRDE-3A) rotator were used, each equipped with an Au-RDE.

## Results and Discussion

Figure 1 shows exemplary SEM images of the pristine film (Figure 1a) and the activated catalyst after electrochemical investigations under OER conditions (Figure 1b) on the Au-RDE surface. The electrochemical OER protocol is provided in SI-Section 1.4. It contains ten CV cycles, required to transform the selenide pre-catalyst to the catalytically active oxyhydroxide species at the catalyst surface. The thin nanoparticulate film consists of grains in the sizes of tens to hundreds of nanometers. While the morphology of the film is similar before and after electrochemical activation, EDX analyses show a significant increase in the atomic Ni:Se ratio. As-deposited (pristine) films have an atomic Ni:Se ratio of 3:2, while after activation the Se content is strongly decreased (to ≈ 25% of pristine content, as shown in Figure S2 in the SI). Accompanied by a dramatically increasing oxygen content (oxygen was not even detected in the pristine film), these findings indicate the transformation of the as-deposited nickel selenide to a nickel oxide/oxyhydroxide, in agreement with previous studies.^27–29^

**Figure 1:**
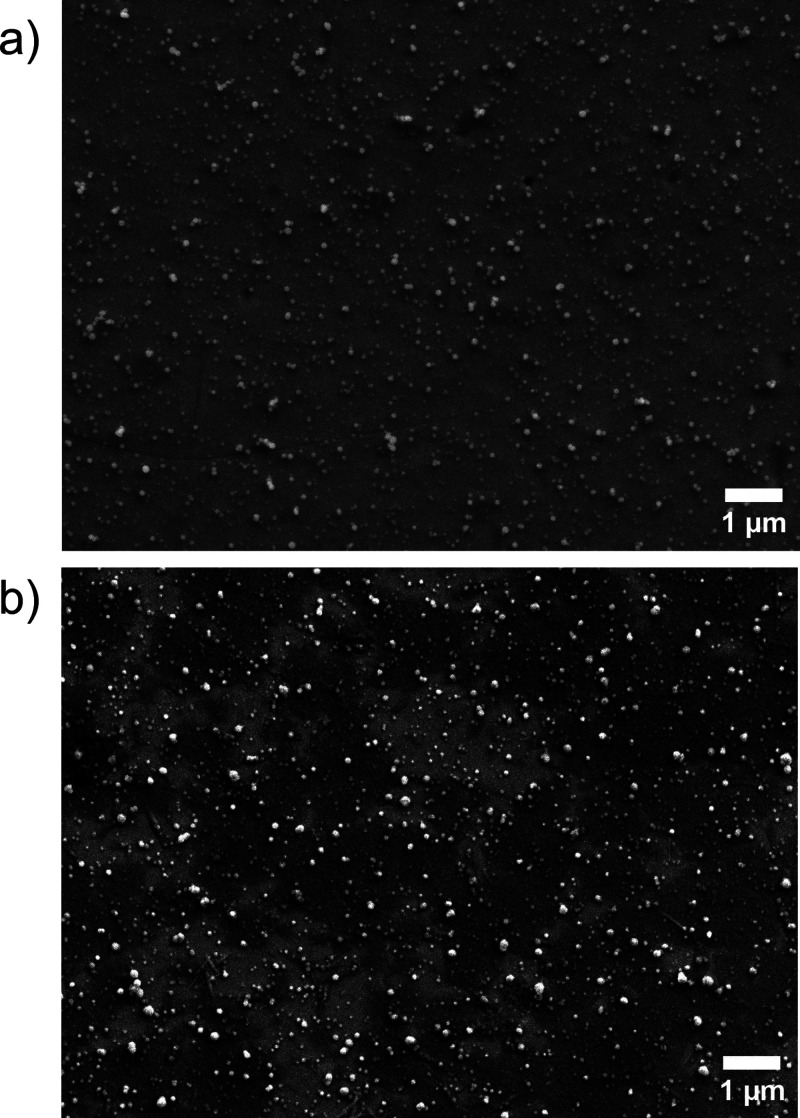
SEM images of the electrodeposited catalyst material a) in the pristine state and b) after electrochemical activation.

Figure 2a) shows the 10^th^ cycle of electrochemical activation at rotation rates of 1600, 2500, and 4000 rpm on Au-RDEs. In the previous nine CV cycles, the catalyst was continuously activated (Figure S3) originating from the well-known transformation of nickel selenides into oxide/oxyhydroxide species. To ensure a high catalytic activity of the films, we focus on the Tafel analysis of the 10^th^ CV cycle. Furthermore, the kinetic current density was calculated according to Equation S2 using the limiting current calculated from the Levich equation (Equation S3) to limit the influence of mass transport processes on the extraction of the kinetic parameters. In the case of the herein-presented experiments, Tafel slopes extracted from experimental current density *j*_geo_ from CVs did not differ significantly from those extracted from *j*_k_ plots (see Figure S4). This indicates that the *j*_geo_ in this work is not limited by the reactant (hydroxide) concentration.^1^ The kinetic current density *j*_k_ in the CV curves in Figure 2a) increases at high OER overpotentials with rotation speed where the current response is dominated by mass transport processes.^2,21^

**Figure 2:**
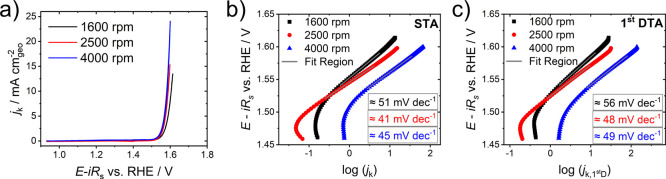
a) Anodic forward scans of CV curves showing the kinetic current density *j*_k_ of the 10^th^ CV cycles in 0.1 M KOH + 1.0 mM Fe­(NO_3_)_3_ recorded at 2 mV s^–1^ for rotation speeds of 1600, 2500, and 4000 rpm of electrodeposited nickel selenide (pre-)­catalyst films; b) STA and c) DTA plots extracted from CV curves in a).

Figures 2b) and c) show the Tafel plots extracted from the CV curves presented in Figure 2a). Tafel analyses were performed in a 50-mV-potential window (ca. one decade of *j*_k_) where the transfer coefficient *α* was constant (max. standard deviation (StD) ≤ 3%, see Figure S5) based on the procedure reported by Khadke et al.^1^ A larger potential window for Tafel analysis is not justified for our system, as the transfer coefficient α would vary significantly (see Figure S5). Moreover, the surface coverage of hydroxide on the catalyst may change and a potential dependent chemical transformation of the catalyst material may not be excluded, which would result in additional currents not associated with OER. In cases where these aspects can be safely excluded, mass transport correction of the current can be done, as reported in several previous manuscripts and textbooks.^15,17,35^

Both, STA and DTA plots show deviating values when the rotation speed is changed. The highest Tafel slopes were found for 1600 rpm whereas 2500 rpm showed the lowest slopes for both approaches. The Tafel slopes using the STA method were determined by both, individual and automated analysis using a JavaScript code (see SI-Section 1.6 for details). Both methods yielded identical values. The Tafel slopes calculated applying DTA (1^st^ derivative of the current response) were higher at all rotational speeds.

Notably, a wide range of Tafel slopes are reported frequently for a similar catalyst system, e.g., for nickel-selenide-based OER electrocatalysts, Tafel slopes from 30 to 120 mV dec^–1^ were reported in recent years by various groups.^26^ The reason for this variety of Tafel slope values for a similar system is likely due to the large number of different experimental and data analysis techniques used.^2,36–38^ Thus, benchmarking experimental Tafel slope values using literature reports is a complex task.

Voltammetry approaches are by a large margin the most used techniques for the determination of Tafel slopes. However, the potential sweep in CV measurements leads to dynamic changes at the solid/liquid interface, such as adsorption/desorption phenomena and altered electrostatic interactions, which result in additional currents potentially distorting the Tafel analysis. A possible alternative to estimate Tafel slopes is applying chronoamperometry to extract steady state current values at different potentials and use these to extract kinetic parameters. Several works have suggested that this approach can lead to (apparent) higher exchange current densities and lower Tafel slopes in comparison to those extracted from CV experiments.^3,12,14,19,12^,

Thus, we applied a series of chronoamperometric experiments starting at a potential around the nickel oxidation peak, increasing the potential in 10-mV steps from a CA experiment to the next one (see SI experimental section for details). To allow equilibration of the system and achieve steady state conditions,^14^ the current response was extracted 5 min after starting the CA measurement. Before performing Tafel analyses, the kinetic current densities *j*_k_ were calculated and the potential was *iR*-corrected using the solution resistance *R*_s_ (Figure S6).

To ensure direct comparability of the results of these chronoamperometric experiments with the previous potentiodynamic studies, the catalyst was activated by the same procedure as before prior to the CA measurements (10 CV activation cycles, see SI-Section 1.4 for details).

Figure 3 shows the Tafel plots from the CA approach at rotation speeds of a) 1600, b) 2500, and c) 4000 rpm. For all experiments, the same potential range was used (ca. 1.54–1.57 V vs. RHE, *iR*-corrected) where R^2^ > 0.996 (covering > one decade of *j*_k_). However, the width of the potential window is ca. 20 mV smaller than in the standard and 1^st^ derivate Tafel analyses from the CV experiments in Figure 2. Thus, we additionally provide the standard and 1^st^ derivate Tafel analyses in a 30-mV-window with constant *α* in Figure S7 which yield similar values compared to the Tafel slopes in the 50-mV-window at all three rotation rates (Figure 2).

**Figure 3:**
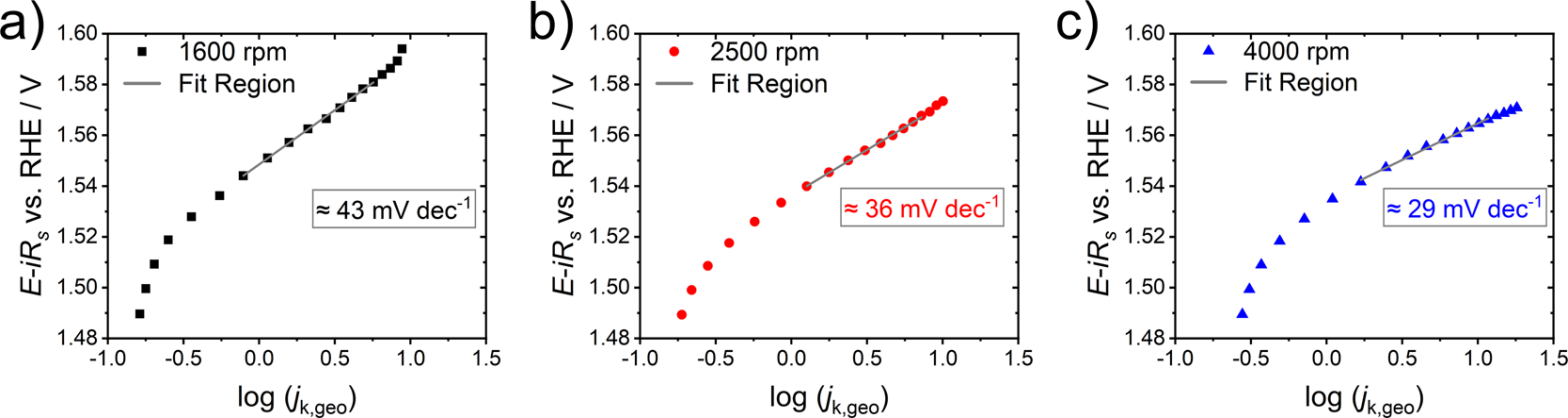
Chronoamperometric Tafel analyses were recorded in 10 mV steps (iR-correction afterwards) in 0.1 M KOH + 1.0 mM Fe­(NO_3_)_3_ at rotation speeds of a) 1600, b) 2500, and c) 4000 rpm of electrodeposited nickel selenide (pre-)­catalyst films.

The Tafel slopes decreased significantly with the used rotation rate during the RDE experiments. Whereas 43±1 mV dec^–1^ was obtained at 1600 rpm, the Tafel slope decreased to 29±1 mV dec^–1^ at 4000 rpm. The latter value is in good agreement with the Tafel slope reported by van der Heijden et al. for a nickel­(iron) oxyhydroxide OER electrocatalyst extracted from CV experiments in a small potential range at low current densities.^2^ Furthermore, values of around 30 mV dec^–1^ are the lowest reported in the literature for Ni-based OER catalysts,^5,30^ indicating that the rate-determining step at these catalysts involves a single electron transfer under the applied conditions.

The lower Tafel slopes obtained by the CA method are neither due to changes of the catalyst occurring throughout the experiment nor the longer duration in comparison to the CV approach. This becomes clear from Figure S8, which shows that the same Tafel slope is obtained for the CV-based characterization at 4000 rpm before or after exposing the catalyst to CA experiments. Thus, the longer experimental time of the CA experiment did not cause a further activation of the catalyst, which would have resulted in lower Tafel slopes. Instead, the data suggest that the catalyst activity remained constant throughout the CA experiment, in agreement with previous reports on nickel selenide-derived OER electrocatalysts.^26,39,40^

Figure 4 summarizes the results of Tafel analyses when conducting either the CV or the CA methods on the nickel selenide films, which were transformed into an oxide/oxyhydroxide species. Their distinct differences underline the utmost importance of reporting the experimental conditions alongside the Tafel slope values, especially concerning the rotational speed; best in the form of data and associated metadata in a research data management system.^41^ Furthermore, it is advised to include automated data processing to reduce effort for researchers and obtain consistent user-independent data analysis to complement the evaluation made by experienced users. This applies to both potentiodynamic (CV)^2,5^ and potentiostatic (CA), experiments.

**Figure 4:**
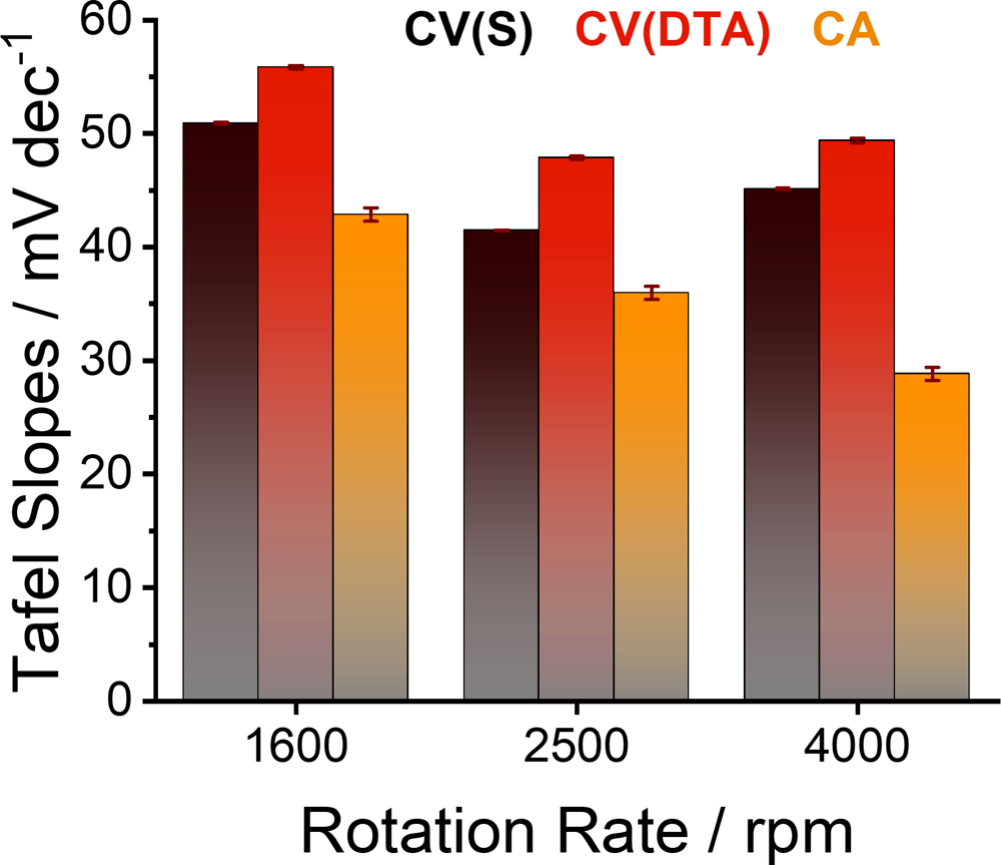
Summarizing bar chart of extracted Tafel slopes from CV experiments (STA, CV­(S), and DTA, CV­(DTA)) and chronoamperometry experiments (CA) at RDE rotation speeds of 1600, 2500, and 4000 rpm of the electrodeposited nickel selenide (pre-)­catalyst films. At all rotational rates, the Tafel slopes calculated via the CA approach were significantly lower than the CV methods, and the Tafel slopes from the CA approach decreased significantly with increasing rotation speed.

Thus, we suggest extracting the Tafel slope of an electrocatalytic system via chronoamperometric experiments at the highest rotation rates that are experimentally accessible without catalyst detachment and without violating laminar flow at the RDE. If the microscopic and geometric surface areas of the catalyst film are in the same order of magnitude, laminar flow conditions can usually be met for rotation rates of up to 10000 rpm assuming the electrode to be a flat rotating cylinder (*d* ≥ 1 mm)^15^ in an aqueous electrolyte of low viscosity^42^ (kinematic viscosity *ν* ≈ 0.01 cm^2^ s^–1^).^43^

Furthermore, kinetic data extracted from experiments using membrane electrode assemblies (MEA) showed improved quality towards those extracted from RDE experiments due to the lower influence of mass transport limitations, e.g., bubble and nanobubble formations on top and inside the catalyst layers.^44–47^ However, those comparative studies of MEA and RDE found that the general trends gained from RDE experiments are still valid in MEA experiments when investigating different electrocatalysts, while the absolute values may differ.^44,45^

## Conclusions

We wish to point out that the accuracy of mechanistic and kinetic insights achievable from Tafel slopes for general electrocatalytic experiments are frequently overestimated and more caution should be put on selecting how to appropriately obtain and interpret data for the specific system studied. This is not only due to boundary requirements implied in the Butler-Volmer-formalism, but also due to the influence of used experimental parameters, such as scan rate, rotation speed, cell geometry, and working electrode size and morphology.^2,6–8,12–14,18^ Using electrodeposited nickel-selenide-based OER electrocatalysts we found that chronoamperometric experiments at the highest rotation speed produced the lowest Tafel slope, hinting that the steady state and mass transport conditions are satisfied for Tafel analysis and potential extraction of further kinetic parameters, e.g., exchange current densities or charge transfer coefficients.

The fact that a homogeneously accessible electrode is used is usually implicit in Tafel analysis. We achieved this by electrodeposition of our non-porous catalyst to ensure a homogenous and dense distribution of catalytically active sites on our macroscopic, non-porous electrode. If non-homogeneously accessible electrodes are to be considered, for instance, in the case of smaller,^48^ porous,^49^ fractal,^50^ or nanoparticle decorated electrodes,^51^ then additional care is required for appropriate data analysis, as highlighted and discussed in detail in previous works by Compton and others.^35,52^

Notably, Tafel plots are potentially useful for qualitatively determining the activation trend of the catalyst material during ongoing CV cycles,^53^ even if they may not allow for the extraction of kinetic parameters, such as exchange current densities or charge transfer coefficients, quantitatively for many experimental cases.

## Supplementary Material



## Notes

Detailed experimental section with the full electrochemical protocol, additional electrochemical activity data, SEM and EDX data, and plots of the charge transfer coefficient *α* to determine the potential ranges for performing Tafel analysis (PDF)

## ∥

Technische Universität (TU) München, Campus Straubing für Biotechnologie und Nachhaltigkeit, Uferstraße 53, 94315 Straubing, Germany

## ABBREVIATIONS

OER: oxygen evolution reaction
RDE: rotating disk electrode
CV: cyclic voltammetry
CA: chronoamperometry
ECSA: electrochemical surface area
STA: standard Tafel analysis
DTA: differential Tafel analysis
MEA: membrane electrode assemblies
