# Supplementary material for: The Crucial Role of Rotation Speed on the Determination of Tafel Slopes of Electrocatalysts in Rotating Disk Electrode Experiments
Source: ACS Electrochem. 2025 Jul 24;1(10):2149–55. doi: 10.1021/acselectrochem.5c00210 (PMC12498423; doi:10.1021/acselectrochem.5c00210)
Supplement: Supplementary file 1 [file ec5c00210_si_001.pdf]

Supporting Information (SI): The Crucial Role of Rotation Speed on the Determination of Tafel Slopes of Electrocatalysts in Rotating Disk Electrode Experiments

Felix Hiege,[a] Luca Marie Sicking,[a] Kannasoot Kanokkanchana,[a] Paolo Cignoni,[b] Victor Dudarev,[c] Alfred Ludwig,[c] and Kristina Tschulik[a,b*]

[a]Ruhr-Universität Bochum, Lehrstuhl für Analytische Chemie II, Universitätsstraße 150, 44801 Bochum, Germany
[b]Max-Planck-Institut für nachhaltige Materialien GmbH, Max-Planck-Straße 1, 40237 Düsseldorf, Germany
[c]Materials Discovery and Interfaces, Institute for Materials, Ruhr University Bochum, Universitätsstraße 150, 44801 Bochum, Germany
*Corresponding author: Kristina.Tschulik@rub.de

1. Experimental Section:

1.1 Electrochemical setup:

All electrochemical measurements were conducted using a BioLogic VSP-300 potentiostat. As a counter electrode (CE), a C rod (*d* = 6 mm), and as a reference electrode (RE), a homemade Ag|AgCl|3 M KCl were used. To guarantee an accuracy of at least ± 5 mV, the potential shift of the RE was checked before and after every experiment. For the measurements in alkaline electrolytes, a commercial Hg|HgO RE (*ALS* RE61-AP) filled with 0.1 M KOH in the body was used. Additionally, during the OER investigations in alkaline electrolytes a Pt wire (*d* = 0.25 mm, Alfa Aesar; 99.95 % metal-based) and a 100-pF bypass capacitor were added to the RE to suppress the influence of stray capacitances at high frequencies during the electrochemical impedance measurements. For all electrochemical measurements, a three-neck electrochemical cell made of quartz glass was used. RE and CE were located in separate compartments connected to the main body via frits.

As WE Pine Research E6R1 ChangeDisk polytetrafluoroethylene (PTFE) RDE with exchangeable gold disks (*d* = 5 mm) were used for the experiments with rotation speeds of 1600 and 2500 rpm. The outer diameter (OD) was 15 mm and the electrode was mounted in an MRS rotator (AFMSRCE, Pine Research). Since the Pine rotator only allows rotation speeds up to 3000 rpm, experiments at 4000 rpm were conducted with a rotator by *ALS* (RRDE-3A) using a gold disk of 3 mm diameter (011171, ALS) with an OD of 12 mm. Notably, the ratio of the active electrode diameter to the diameter of the insulating shroud of the electrode body can affect the mass flux and with this current response[1] and the small electrode diameter of the ALS RDE concerning the shroud size is not ideal. Irrespective of that, Figure S1 shows that the Tafel slopes determined using the CA approach at 1600 rpm and 2500 rpm with the ALS RDE are similar to those observed at the Pine RDE. However, the Tafel slopes extracted from CV are higher for the Pine than the ALS RDE for rotation rates of 1600 rpm (≈27 %) and 2500 rpm (≈7 %).

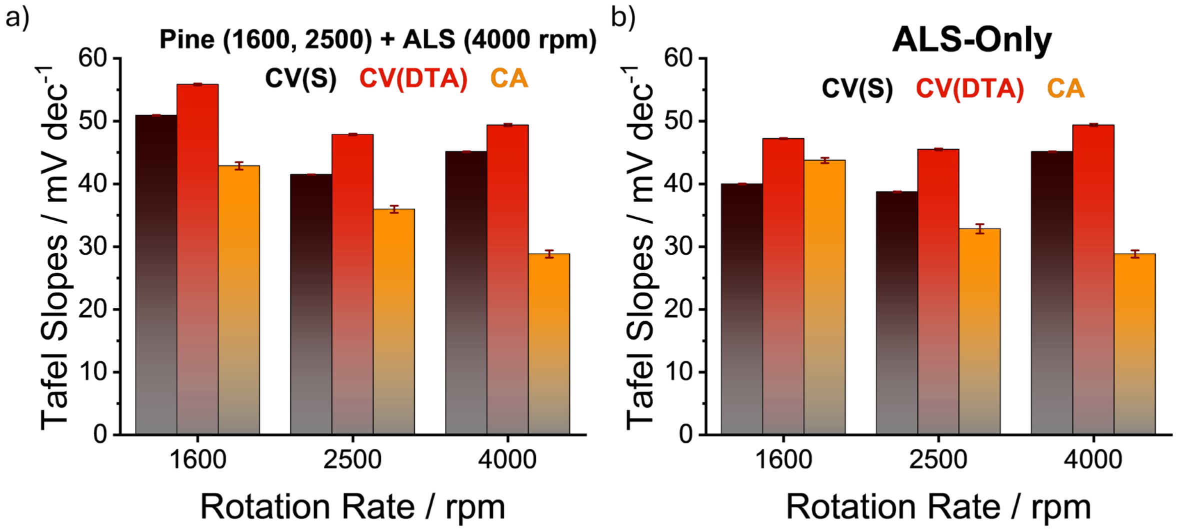

Figure S1. a) Bar diagram of extracted Tafel slopes from CV experiments (standard Tafel analysis, CV(S), and 1st derivative Tafel analysis, CV(DTA) and chronoamperometry experiments (CA) at RDE rotation speeds of 1600 rpm, 2500 rpm on Pine RDE, and 4000 rpm on ALS RDE of electrodeposited nickel selenide (pre-)catalyst films (same plot as Figure 4 in main article); b) Bar diagram of extracted Tafel slopes from CV experiments (standard Tafel analysis, CV(S), and 1st derivative Tafel analysis, CV(DTA) and chronoamperometry experiments (CA) at RDE rotation speeds of 1600, 2500, and 4000 rpm all on ALS RDE.

This latter observation can be rationalized by the fact that macro electrodes of smaller diameter have a larger contribution of radial diffusion than bigger ones. Thus, the current response resulting from this radial diffusion adds to the classically assumed current from planar diffusion at ideal macro electrodes. These so-called “edge effects” are more relevant for larger diffusion layer thicknesses and thicker stagnant (Prandtl) layers at the RDE at lower rotation speeds.[2] The edge effects cause the observed difference between the differently sized electrodes at lower rotation speeds in Figure S1. Since the thickness of the diffusion and Prandtl layer decreases with increasing rotation speed, the deviation of the Tafel slopes determined using RDEs of different sizes is smaller at 2500 rpm.

Summarized, we highlight that the geometrical characteristics of the set-up used should be considered when comparing Tafel slopes. Furthermore, we emphasize that, whenever possible, the use of electrodes with a large diameter (> 4 mm)[3] and operating at the highest possible rotation speed should be preferred.[2]

1.2 Electrochemical Cleaning of Au-RDE:

The Au-RDE was electropolished in a series of chronoamperometric (CA) and cyclovoltammetric (CV) experiments in 0.5 M H_2SO_4. First, a 30 s CA at 3.0 V vs. Ag|AgCl|3 M KCl was conducted to oxidize the gold surface. Next, the gold oxide was removed chemically by immersing the Au-RDE in concentrated HCl for a few seconds. This step was repeated once. Afterward, a CV experiment of 20 cycles at a scan rate of 100 mV s^{-1} in a potential window of -0.1 to 1.8 V vs. Ag|AgCl|3 M KCl (start/end potential 0.2 V) was performed, followed by another CA at 3 V vs. Ag|AgCl|3 M KCl for 30 s and removal of the gold oxide by immersing in concentrated HCl.

Before the electrodeposition of nickel selenide, 20 CV cycles in a potential window of -0.1 to 1.45 V vs. Ag|AgCl|3 M KCl (start/end potential 0.2 V) at a scan rate of 100 mV s^{-1} in 0.5 M H_2SO_4 on Au-RDE were monitored to i) clean its surface by multiple oxidation and reduction of the gold surface and ii) estimate the microscopic area A_m based on the gold reduction peak of the last CV cycle and the assumption of a gold oxide monolayer on a polycrystalline gold surface (482 µC/cm^2).[4] Roughness factors in the conducted experiments, determined by dividing A_m by the geometric area A_{geo}, range between 1.1 and 1.5.

Before this cleaning procedure previously deposited NiSe films were removed from the Au-RDE by applying a potential of 1.0 V vs. Ag|AgCl|3 M KCl for 30 s in 0.5 M H_2SO_4,

1.3 Electrodeposition of nickel selenides:

The electrodeposition procedure of nickel selenides was selected based on the procedure of Cao et al.[5] The precursor solutions were 100 mM $NiSO_4 \cdot 6H_2O$ (Sigma Aldrich, ≥99.99 % trace metal basis), 100 mM SeO_2 (Sigma Aldrich, 99.9 % metal basis), and 250 mM K_2SO_4 (Supleco) were prepared using ultra-pure water (≤0.055 µS cm^{-2} at 25 °C). For the electrodeposition of nickel selenide, 5 mL of each stock solution was mixed with 35 mL of ultra-pure water to get a 50 mL solution with concentrations of 10 mM $NiSO_4$, 10 mM SeO_2, and 25 mM K_2SO_4 (pH 2.5). Afterward, the solution was deoxygenated under Ar-flow for at least 20 minutes.

Each electrodeposition was performed under coulometric control in chronoamperometric experiments

Until a charge of 76 mC cm^{-2} was passed, using the three-electrode setup described above and a deposition potential of -0.79 V vs. Ag|AgCl|3 M KCl. Additionally, a double junction containing 25 mM K_2SO_4 was used to prevent chloride contamination from the RE.

1.4 Electrochemical Protocol to Investigate OER Performance in Alkaline Electrolytes:

The OER catalytic performance of the electrodeposited (pre-)catalysts on RDEs was investigated in 0.1 M KOH (Sigma Aldrich) with intentionally added 1.0 mM $Fe(NO_3)_3$ (Sigma Aldrich), similar to the procedure developed by the Boettcher group.[6] Tiny amounts of iron ions (on the ppb scale) in the electrolyte are known to enhance the catalytic activity of nickel-based OER electrocatalysts significantly.[7] Thus, we intentionally added a defined amount of iron ions to the KOH electrolyte to avoid the influence of unintentional iron contamination and ensure comparability between the experiments. After the addition of 1.0 mM $Fe(NO_3)_3$ and 24 h of resting, the $Fe(OH)_3$ precipitants were filtered off using a 3 mL syringe (BD 3ml Syringe, Luer-Lok™ Tip) and a 0.2 µm PTFE filter (Acrodisc® CR 25 mm Syringe Filter). Using ICP-MS, ≈14 µmol/L in the KOH solution was found in a previous study of our group, following the same preparation method.[8] With this approach of an intentionally increased iron concentration in the electrolyte, the influence of unintentionally incorporated iron contaminations on the catalytic performance of the electrodeposited catalysts becomes negligible.

The electrochemical protocol consisted of a sequence comprising nine steps, containing open circuit potential (OCP) determination periods, electrochemical impedance spectroscopy (EIS) measurements at a constant dc potential, and cyclic voltammetry (CV) experiments:

1. OCP, 5 min
2. EIS-0 @ OCP
3. CV-period 1: 5 cycles @ 2 mV/s, 0 – 0.8 V vs. Hg|HgO|0.1 M KOH
4. OCP, 1 min
5. EIS-1 @ 1.6 V vs. RHE → 0.67 V vs. Hg|HgO|0.1 M KOH
6. OCP, 1 min
7. CV-period 2: 5 cycles @ 2 mV/s, 0 – 0.8 V vs. Hg|HgO|0.1 M KOH

8. OCP, 1 min
9. EIS-2 @ 1.6 V vs. RHE → 0.67 V vs. Hg|HgO|0.1 M KOH

The chronoamperometric experiments were conducted after these nine steps starting with an OCP determination for 1 minute followed by the first CA at 0.56 V for 5 minutes. The current recorded after 5 minutes was used to calculate j_k for the CA Tafel plots. CA experiments were conducted in steps of 10 mV (final potential 0.73 V). Before each CA measurement, the OCP was monitored for 1 minute.

The potentiostatic impedance spectroscopy measurements (PEIS) were conducted with an amplitude of 7.1 mV (rms) in a frequency range from 200 kHz to 0.05 Hz.

All electrochemical potentials were converted from Hg|HgO|0.1 M KOH to RHE scale using Equation (S1)

$$E_{RHE} = E_{(\mathrm{Hg|HgO|0.1\ M\ KOH})} + (0.059 \cdot pH) + 0.164\ \mathrm{V} \quad \text{(S1)}$$

1.5 Analysis of Electrochemical Data:

Tafel slopes were extracted from the forward scans in the 5th cycle of the 2nd CV period (10th cycle overall) using *OriginPro 2021b*. The Tafel slopes were estimated from the Tafel plots (*E* vs. log(*j*)) in a potential range of 50 mV where the charge transfer coefficient alpha was constant to < 3 %. The R^2 value of the linear fits was >0.996 in all experiments.

Analysis of the PEIS data at 1.6 V vs. RHE to determine the solution resistance R_s used to calculate the iR-compensated from the experimental data was performed using *RelaxIS 3* (rhd instruments). For data processing, a Z-HIT algorithm (1 kHz to 0.1 Hz range) was used, before the equivalent circuit model (R-C-R Randles circuit) was applied to the experimental data.

The kinetic current i_k was calculated using the Koutecký-Levich correction (Equation (S2)).

$$i_{\mathrm{k}} = \frac{i i_{\mathrm{L}}}{(i_{\mathrm{L}} - i)} \quad \text{(S2)}$$

In Equation (S2), i is the experimental current and i_L is the limiting current calculated from the Levich equation[2,9]:

$$i_{\mathrm{L}} = 0.62\ D^{2/3}\ \omega^{1/2}\ \nu^{-1/6} \quad \text{(S3)}$$

In Equation (S3), a diffusion coefficient of OH^- ions in 0.1 M KOH ($2.646 \cdot 10^{-5}$ cm^2 s^{-1})[10] was used for *D*, the rotation speed ω was selected depending on the experimental conditions either 167.55, 261.80, or 418.88 Hz (corresponding to 1600, 2500, 4000 rpm), and the kinematic viscosity of a 0.1 M KOH solution (0.01 cm^2 s^{-1}, estimated to be similar to an aqueous solution)[11] was used for ν. For the 1st DTA analysis, differentiation was performed after smoothing with a 20-point adjacent averaging procedure.

1.6 Automated STA using JavaScript

Automated STA was developed as a web application primarily using client-side *JavaScript* code written by Dr. Victor Dudarev in the group of Prof. Dr. Alfred Ludwig. The automated STA is intended to be implemented as an internal standard procedure within the CRC 247 RDMS.[12]

The program extracts the experimental potential and current responses of a CV experiment from the raw data file (TXT and CSV formats accepted). The program identifies the number of CV cycles included in the data file and asks the user to select the cycle of interest for analysis. Further, the user has to provide the following parameters: rotation speed of the experiment, solution resistance (determined from EIS experiment), the temperature of the experiment, the radius of the working electrode, identity and concentration of the (alkaline) electrolyte including the reference electrode system for conversion of potentials to RHE scale, and the potential window for the Tafel analysis (50 mV in this work).

Using these parameters, the program automatically performs ohmic drop compensation using the solution resistance of the experimentally applied potential and converts it to the RHE scale following equation (S1). Furthermore, it calculates the kinetic current i_k according to SI-Section 1.5, which is used to derive the plot of the potential-dependent charge transfer coefficient α via three steps: 1) calculating the $\log_{10}$ of i_k, 2) calculating the first derivative of $\log_{10}(i_k)$ concerning the *iR*-corrected potential (converted to RHE scale), and 3) dividing the values obtained from steps 1) and 2) by $f = F/RT$, with the Faraday constant F, the ideal gas constant R, and the temperature of the experiment T.

The program identifies in the plot of α against the applied (*iR*-corrected) potential the region in which α (boundary conditions: $0 < \alpha < 1$ and maximum in α plot against the applied potential in the potential region past the Ni oxidation peak, meaning >1.47 V vs. RHE) is constant to < 3 % by moving averaging of α in the user-defined potential window width (e.g., 50 mV in this work). The program gives the potential range as an

output in which the standard deviation (StD) of the average a is minimized, e.g., 1.54 – 1.59 V vs. RHE in the case of our CV experiment at 1600 rpm (see Figure S5a)).
This potential range is used by the program to perform the STA of the applied (iR-corrected) potential against $\log_{10}(i_k)$ and gives the visualized Tafel plot as well as the numbers of the Tafel slope, its error, and the R^2 from the linear fit as the final output.

1.7 SEM/EDX Investigations:

Scanning electron microscopy (SEM) investigations on Au-RDEs were performed using a high-resolution scanning electron microscope *JSM-IT800* (JEOL) at 3 kV. An Oxford *Ultim Max Silicon Drift Detector* (SDD) at 10 kV was used as primary detector to monitor energy dispersive X-ray (EDX) spectra. Data processing and analysis of the EDX spectra was performed using Oxford *AZtecLive* and errors of the compositional analysis varied between 3% and 5%.

2 Supplementary Figures:

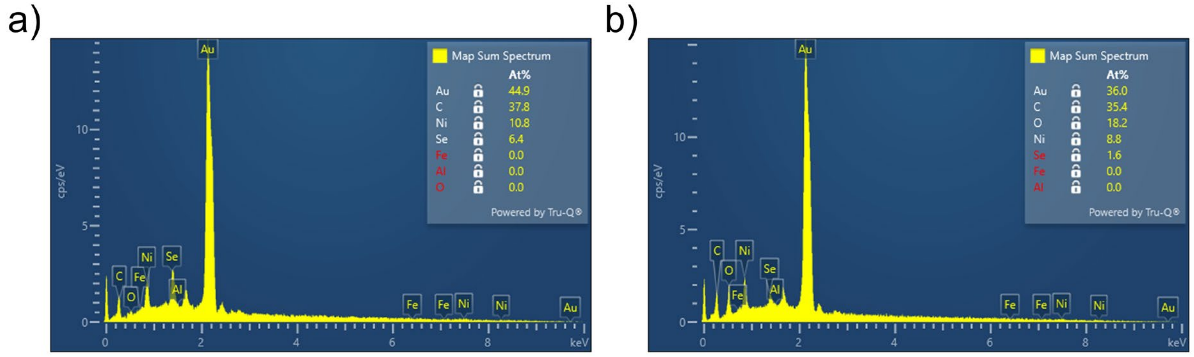

Figure S2. EDX spectra of the electrodeposited catalyst a) in the as-deposited state and b) after electrochemical characterization.

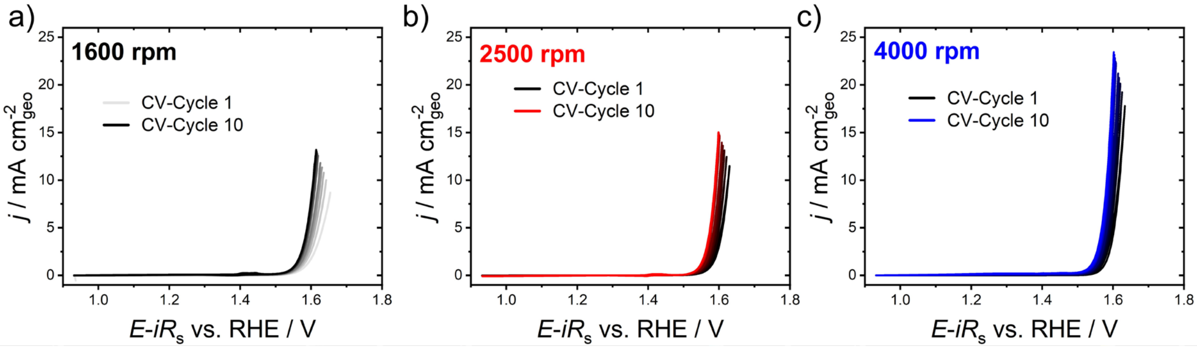

Figure S3. Anodic forward scans of CV experiments of the electrochemical activation of electrodeposited nickel selenide films in 0.1 M KOH + 1.0 mM $Fe(NO_3)_3$ at rotation rates of a) 1600, b) 2500, and c) 4000 rpm of the electrodeposited nickel selenide (pre-)catalyst films. All experiments exhibit an activation trend from the first to the 10th CV cycle.

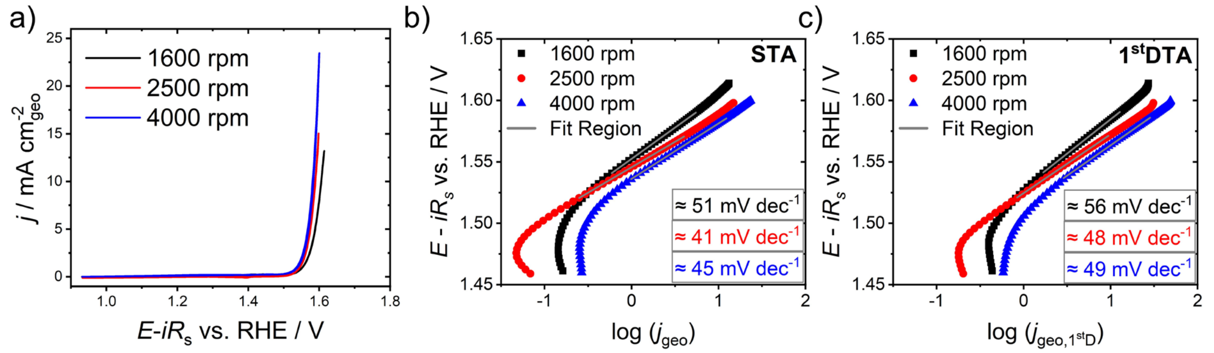

Figure S4. a) Anodic forward scans of CV experiments showing the geometric current density j_{geo} of the 10th CV cycles in 0.1 M KOH + 1.0 mM $Fe(NO_3)_3$ recorded at 2 mV s^{-1} for rotation speeds of 1600, 2500, and 4000 rpm of electrodeposited nickel selenide (pre-)catalyst films; b) STA and c) DTA plots extracted from CV curves in a).

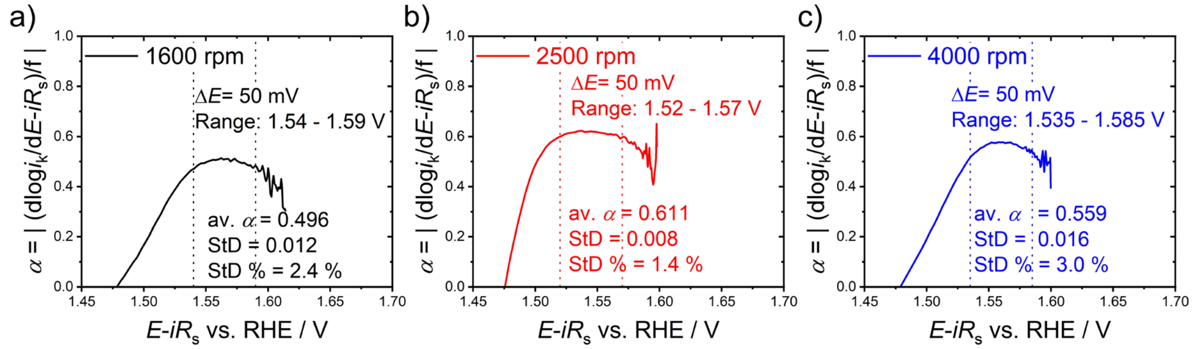

Figure S5. Plots of the calculated charge transfer coefficient α against the iR-corrected potential produced according to Khadke et al.[13] from the CV curves in Figure 2 a) at rotation rates of a) 1600, b) 2500, and c) 4000 rpm of electrodeposited nickel selenide (pre-)catalyst films. Plots were used to identify a 50-mV window (dashed lines) in which alpha was constant to ≤ 3 % for accurate Tafel slope extraction.

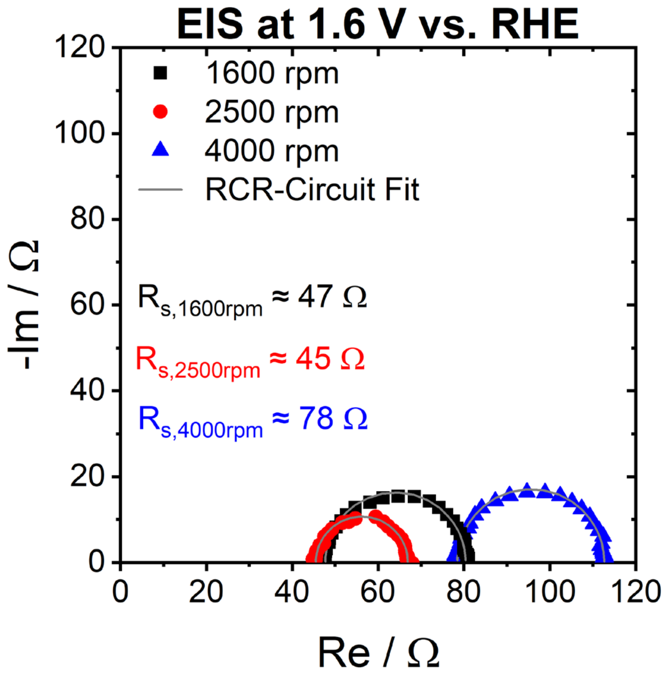

Figure S6. EIS spectra at 1.6 V vs. RHE (OER range) were used for the extraction of the solution resistance R_s at each rotation rate. R_s values were determined by conducting an R-C-R fit using *Relaxis 3* software (see experimental section above) as 45 Ω at 1600, 47 Ω at 2500, and 78 Ω at 4000 rpm. Noteworthy, the experiments at 4000 rpm were conducted at an Au-RDE by ALS, whereas those at 1600 and 2500 rpm were performed using a Pine Rotator (see experimental section above).

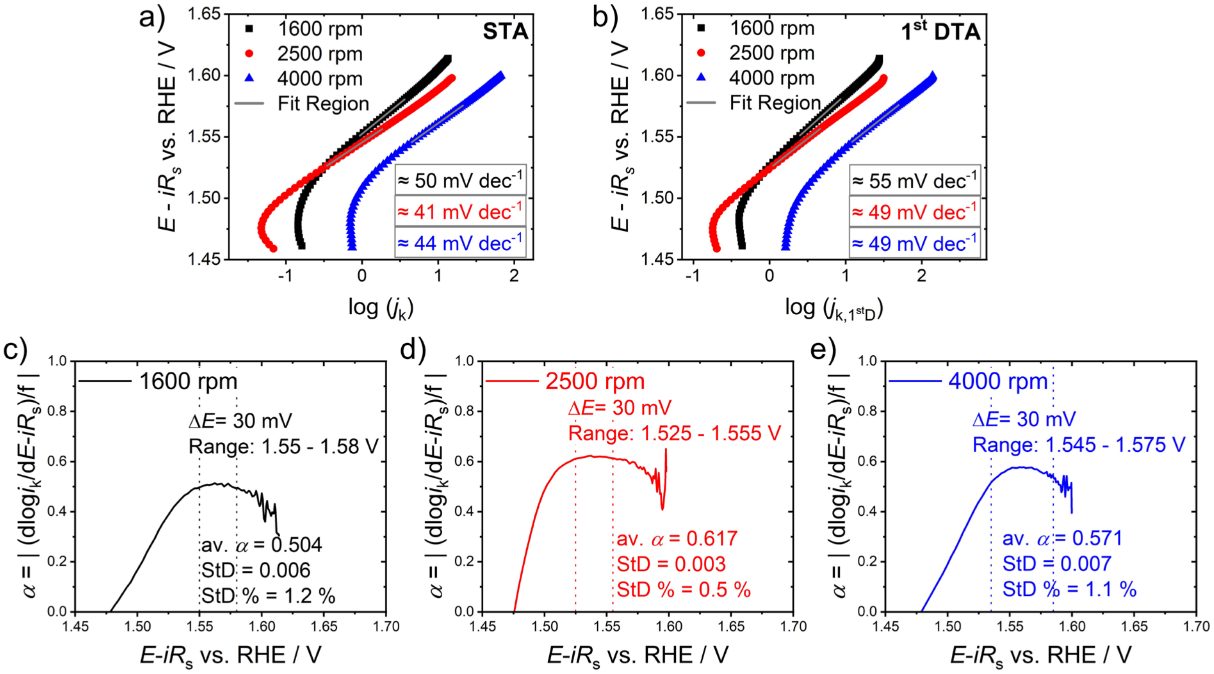

Figure S7. a) STA and b) DTA plots extracted from CV curves in Figure 2a) in the main text in a 30-mV potential window (dashed lines); Plots of the calculated charge transfer coefficient alpha against the iR-corrected potential produced according to Khadke et al.[13] from the CV curves in Figure 2 a) at rotation rates of a) 1600, b) 2500, and c) 4000 rpm of electrodeposited nickel selenide (pre-)catalyst films. Plots were used to identify a 30-mV window in which alpha was constant to < 3 % for accurate Tafel slope extraction.

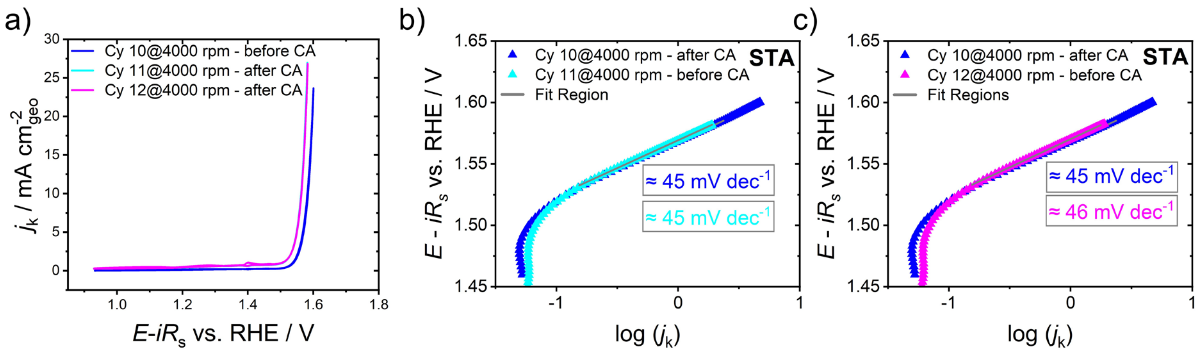

Figure S8. a) Anodic forward scans of CV experiments with the kinetic current density j_k of the 10th, 11^{th,} and 12th CV cycles in 0.1 M KOH + 1.0 mM $Fe(NO_3)_3$ recorded at 2 mV s^{-1} recorded for rotations speeds of 4000 rpm of an electrodeposited nickel selenide (pre-)catalyst film after the determination of Tafel slope using the CA approach (see SI-section 1.4). In addition to the ten CV cycles run in the electrochemical protocol, the CV cycles are named Cy 11 and 12; STA extracted from Cy 11 (b) and Cy 12 (c) show the same Tafel slop as determined for Cy 10. This indicates that the observed lower Tafel slopes from the CA approach are not caused by ongoing material transformation and with this activation due to the longer timescale of the experiment.

References

(1) Levich, V. G. *Physicochemical Hydrodynamics*, 2. ed.; Prentice-Hall international series in the physical and chemical engineering sciences; Prentice-Hall, Englewood Cliffs, NJ, 1962.

(2) Bard, A. J.; Faulkner, L. R.; White, H. S. *Electrochemical Methods:Fundamentals and Applications*, Third edition; Wiley, Hoboken, NJ, USA, Chichester, West Sussex, UK, 2022.

(3) Ngamchuea, K.; Eloul, S.; Tschulik, K.; Compton, R. G. Planar Diffusion to Macro Disc Electrodes—What Electrode Size Is Required for the Cottrell and Randles-Sevcik Equations to Apply Quantitatively? *J. Solid State Electrochem.* **2014**, *18*, 3251–3257. https://doi.org/10.1007/s10008-014-2664-z.

(4) Oesch, U.; Janata, J. Electrochemical Study of Gold Electrodes with Anodic Oxide Films—I. Formation and Reduction Behaviour of Anodic Oxides on Gold. *Electrochim. Acta* **1983**, *28*, 1237–1246. https://doi.org/10.1016/0013-4686(83)85011-7.

(5) Cao, X.; Hong, Y.; Zhang, N.; Chen, Q.; Masud, J.; Zaeem, M. A.; Nath, M. Phase Exploration and Identification of Multinary Transition-Metal Selenides as High-Efficiency Oxygen Evolution Electrocatalysts Through Combinatorial Electrodeposition. *ACS Catal.* **2018**, *8*, 8273–8289. https://doi.org/10.1021/acscatal.8b01977.

(6) Trotochaud, L.; Young, S. L.; Ranney, J. K.; Boettcher, S. W. Nickel-Iron Oxyhydroxide Oxygen-Evolution Electrocatalysts: The Role of Intentional and Incidental Iron Incorporation. *JACS* **2014**, *136*, 6744–6753. https://doi.org/10.1021/ja502379c.

(7) Anantharaj, S.; Kundu, S.; Noda, S. “The Fe Effect”: A Review Unveiling the Critical Roles of Fe in Enhancing OER Activity of Ni and Co Based Catalysts. *Nano Energy* **2021**, *80*, 105514. https://doi.org/10.1016/j.nanoen.2020.105514.

(8) Hiege, F.; Chang, C.-W.; Trost, O.; van Halteren, C. E. R.; Hosseini, P.; Bendt, G.; Schulz, S.; Feng, Z.; Linnemann, J.; Tschulik, K. Morphological Degradation of Oxygen Evolution Reaction-Electrocatalyzing Nickel Selenides at Industrially Relevant Current Densities. *ACS Appl. Mater. Interfaces* **2025.** https://doi.org/10.1021/acsami.5c05381.

(9) Compton, R. G.; Batchelor-McAuley, C.; Dickinson, E. J. F. *Understanding Voltammetry:Problems and Solutions*; Imperial College Press, London, 2012.

(10) Noulty, R. A.; Leaist, D. G. Activity Coefficients and Diffusion Coefficients of Dilute Aqueous Solutions of Lithium, Sodium, and Potassium Hydroxides. *J. Solution Chem.* **1984**, *13*, 767–778. https://doi.org/10.1007/BF00647692.

(11) Vaik, K.; Sarapuu, A.; Tammeveski, K.; Mirkhalaf, F.; Schiffrin, D. J. Oxygen Reduction on Phenanthrenequinone-Modified Glassy Carbon Electrodes in 0.1 M KOH. *J. Electroanal. Chem.* **2004**, *564*, 159–166. https://doi.org/10.1016/j.jelechem.2003.08.024.

(12) Dudarev, V.; Banko, L.; Ludwig, A. An Extensible Open-Source Solution for Research Digitalisation in Materials Science. *Npj Comput. Mater.* **2025**, *11.* https://doi.org/10.1038/s41524-025-01618-1.

(13) Khadke, P.; Tichter, T.; Boettcher, T.; Muench, F.; Ensinger, W.; Roth, C. A Simple and Effective Method for the Accurate Extraction of Kinetic Parameters Using Differential Tafel Plots. *Sci. Rep.* **2021**, *11*, 8974. https://doi.org/10.1038/s41598-021-87951-z.
